# Effects of Green Extraction Methods on Antioxidant and Antimicrobial Properties of Artichoke (*Cynara scolymus* L.) Leaves

**DOI:** 10.17113/ftb.62.03.24.8267

**Published:** 2024-09

**Authors:** Can Turksever, Duygu Benzer Gurel, Aslı Sahiner, Ozlem Çağındı, Ozlem Kizilirmak Esmer

**Affiliations:** 1 Food Engineering Department, Ege University, Bornova, Ankara Street, 35040 Izmir, Türkiye; 2 Food Engineering Department, Manisa Celal Bayar University, 45140 Manisa, Türkiye; 3 Biology Department, Ege University, Bornova, Ankara Street, 35040 Izmir, Türkiye

**Keywords:** green extraction methods, extraction parameters, artichoke leaves, antioxidant properties, antimicrobial properties

## Abstract

**Research background:**

Artichoke leaves, an important waste product of the food industry, have an important antioxidant and antimicrobial capacity. Although there are several studies in the literature to determine their antioxidant and antimicrobial activity, a comparison of green extraction technologies including microwave, ultrasound probe and ultrasound bath methods in relation to the maceration technique has not been performed. Also, several parameters such as the extraction temperature, power, extraction mode and extraction time are important parameters for obtaining targeted compounds in the highest amount. For this reason, we aimed to compare various extraction methods including microwave-assisted extraction, ultrasound-assisted extraction with probe, ultrasound-assisted extraction in a water bath and maceration in terms of extraction parameters for obtaining bioactive compounds from artichoke leaves.

**Experimental approach:**

Microwave-assisted extraction at two different power values, ultrasound-assisted extraction with probe in continuous or pulsed mode with two different extraction times each, ultrasound-assisted extraction in a water bath at two different power values with two different extraction times each and maceration with two different times were used for the extraction. The extraction temperature is an important parameter affecting the thermal degradation of bioactive compounds. We used a constant extraction temperature of 50 °C. Total phenolic and total flavonoid content, antioxidant capacity, phenolic compound profile analysis by LC-QTOF-MS and antimicrobial activity by agar diffusion and broth microdilution methods were determined.

**Results and conclusions:**

The bioactive compounds were found to be significantly affected by the parameters used in each extraction method. The microwave-assisted extraction method was more efficient than the other extraction methods at both power values. This method also required the shortest extraction time. The ultrasound-assisted probe extraction method was the second most efficient method. The type of process, continuous or pulsed, did not affect the results, but shortening the extraction time led to lower results. A longer extraction time of the ultrasound-assisted extraction in a water bath method led to better results, similar to the ultrasound-assisted probe extraction, regardless of the used power. The extracts were highly effective against many opportunistic and pathogenic microorganisms.

**Novelty and scientific contribution:**

This study provides valuable insights into the extraction parameters of different extraction methods to obtain bioactive compounds from artichoke leaves, which could have potential applications in the food and pharmaceutical industries.

## INTRODUCTION

Recently, industrial waste has become a global problem, which has led to the increased adoption of waste management practices. Agro-industrial waste contains bioactive molecules such as fatty acids and phenolic compounds; thus, fruit and vegetable waste is valuable to the food industry (*1*).

In fruit and vegetable processing plants, the generated waste can account for more than 60 % of the amount of raw material. This waste is rich in phenolic compounds, so it is important to evaluate its ecological and economic aspects (*2*). For example, the heart of the unripen flower, which constitutes 30–40 % of the entire plant, can be used to process fresh and canned artichokes, while the remaining outer leaves and stems are waste materials (*3*).

According to the Food and Agriculture Organization (FAO) of the United Nations in 2020, 1 516 955 tonnes/year of artichokes (*Cynara scolymus* L.) are produced in the world (*4*). Artichoke has a high antioxidant capacity and is one of the top fifty foods with a high antioxidant content (*5*). Due to the many functional components, especially the content of minerals, vitamins, inulin and bioactive phenolic compounds in its edible parts, the artichoke is a vegetable with high nutritional value and health-promoting properties (*6*). In addition, artichoke has a significant antioxidant capacity not only in the heart part but also in the leaf parts (*7*). The phenolic compounds obtained from the artichoke leaf extract have been shown to affect the formation of hydroperoxide, which causes oxidative stress, support liver regeneration, protect the heart and have diuretic, hypoglycaemic, cholesterol-lowering, anticarcinogenic, anti-inflammatory and antimicrobial effects. Caffeoylquinic acid derivatives and flavonoids found in artichoke leaf extracts show therapeutic and antihepatotoxic activities (*8*, *9*).

Because of the high value of bioactive compounds, green extraction methods can provide better results for the yield or content of bioactive compounds. Microwave- and ultrasound-assisted extraction techniques have shown tremendous potential as an efficient green alternative to conventional extraction methods in the extraction of natural products. The advantages of these technologies include short process time, low solvent use, low energy consumption, high extract quality and higher extraction yields (*10*, *11*). The ultrasound-assisted technique can be applied in two ways: (*i*) the probe method directly on the sample, and (*ii*) the water bath method indirectly through the walls of the sample container (*12*). The ultrasound-assisted probe method can be applied in two different modes: continuous and pulsed mode. In pulsed mode, the ultrasonic sonicator works internally during the entire extraction process and the cycle time is the sum of the pulse duration and the pulse interval duration. The proportion of the pulse duration in relation to the cycle time is the percentage of duty cycle (*13*, *14*).

The microwave-assisted extraction method, another modern technique, has been widely used in recent years compared to conventional extraction methods. The microwave method can be briefly explained as the disruption of the cell structure by the penetration of volumetric heating due to microwave irradiation (*15*). This technique uses high-frequency electromagnetic microwaves (300–300 000 MHz) to induce heating, acting directly on the molecules through ionic conduction and dipole rotation mechanisms (*16*). Maceration extraction, in which coarse or powdered plants are soaked in different solvents, is associated with some major drawbacks, including insufficient recovery of extracts, long extraction times and high energy consumption (*17*, *18*).

Therefore, there is great interest in green extraction methods that not only provide high yields but are also faster and less energy-intensive due to their environmental benefits. So far, artichoke by-products have been extracted using different extraction methods: maceration (*19*-*22*), ultrasound-assisted extraction (*23*, *24*), maceration and ultrasound-assisted extraction (*25*), maceration and maceration with ultrasound-assisted extraction (*26*), and maceration, ultrasound-assisted extraction and microwave extraction (*27*). However, to the best of our knowledge, there are no studies that have performed a comparative analysis of the influence of different parameters, such as the amount of applied power, the type of process and the extraction time, on the antioxidant and antimicrobial properties of artichoke leaves using different extraction methods, namely microwave-assisted (MAE), ultrasound-assisted extraction with probe (UAEp), ultrasound-assisted extraction in a water bath (UAEwb) and maceration (M).

Therefore, the aim of the present study is to compare both the green and classical extraction methods by applying different parameters such as power, extraction mode and extraction time at constant temperature in the extraction of artichoke leaves, which are an important waste material in the food industry. Accordingly, the effects of different extraction parameters of each method on the antioxidant and antimicrobial activities of the extracts were investigated.

## MATERIALS AND METHODS

### Sample preparation

The leaves of artichokes (*Cynara scolymus* L.) were supplied from a field in Salihli, Manisa in Türkiye on the harvesting day. The waste accumulated after sorting the artichoke hearts was taken to the cold storage of Ege University Food Engineering Department on the same day. The artichoke leaves were separated from the stems and dried for 9 h in a tray dryer (TK-Lab, Eksis, Isparta, Türkiye) at 65 °C under the airflow of 1.5 m/s until 10 % relative humidity was reached. The dried artichoke leaves were then ground in a hammer mill (Series 2000; Brook Crompton, Huddersfield, UK) and passed through a 300 μm sieve. The ground material was extracted in *φ*(ethanol,water)=0.5 and with a *V*(solvent):*m*(solid)=10:1.

### Extraction methods

In all extraction methods, the temperature was standardised to (50±5) °C to prevent the damage of phenolic compounds by high temperatures as it was determined that the optimum temperature for the extraction of bioactive compounds in artichoke leaves was 53.30 °C (*9*) and 53.40 °C (*23*).

In the ultrasound-assisted probe extraction, an ultrasound probe (model UP400St, 200/240V; Hielscher Ultrasonics GmbH, Teltow, Germany) was used to extract artichoke leaves in a pulse process (50 % circle) and a continuous process (100 % circles) in two different time periods of 15 or 30 min. Our preliminary tests showed that the temperature in the continuous application increased with increasing application time. An ice compress was applied around the beaker in which the solution was prepared to standardise the temperature to (50±5) °C. This step was taken to prevent damage to the phenolic compounds by high temperatures.

In the ultrasound-assisted extraction in a water bath, an ultrasonic water bath (model DK 102P, 35 kHz; Bandelin, Berlin, Germany) adjusted to 50 °C was used to extract the artichoke waste. Two power values of 120 and 240 W for 15 and 30 min were applied.

A household microwave device (MW450, 800 W; Kenwood, Havant, UK) was used for the microwave-assisted extraction at two power values of 440 and 800 W. The samples were extracted for 45 and 30 s respectively, which was determined in the preliminary experiments so that the temperature would not exceed (50±5) °C.

In the maceration process, the solutions were macerated at 50 °C for 12 and 24 h at an agitation speed of 3×*g* in a shaking incubator (KS400i; IKA, Königswinter, Germany). After the treatment, the extracts were centrifuged (Eba 8S; Hettich, Tuttlingen, Germany) at 2323×*g* for 10 min at 4 °C. The obtained supernatants were removed and the same procedure was applied twice more to the residue at the bottom of the centrifuge tubes. After extraction, the supernatants were transferred into a 50-mL volumetric flask and stored in dark glass bottles at -87 °C until analysis. The extractions were carried out in two trials and at least two samples were analysed in each trial.

### Total phenolic content

The total phenolic content (TPC) was determined based on the principle of a redox reaction in which the Folin-Ciocalteu reagent is reduced in an alkaline medium and converted to an oxidised form. In the present study, the method of Rodríguez *et al.* (*28*) was adopted with modifications. Accordingly, 250 µL of 0.5 M Folin-Ciocalteu reagent were added to 100 µL of extract and vortexed (Vortex 2; IKA, Königswinter, Germany) for 30 s. Then, 750 μL of 7 % Na_2_CO_3_ solution were added and thoroughly mixed. After standing for 2 h at room temperature in the dark, the absorbance was read at 765 nm on the Multiskan Go microplate spectrophotometer reader (Thermo Fisher Scientific, Waltham, MA, USA). Total phenolic content is expressed in mg gallic acid equivalents (GAE) per g dry mass.

### Total flavonoid analysis

The total flavonoid content (TFC) was determined spectrophotometrically according to Rodríguez *et al.* (*28*) and Kumaran and Karunakaran (*29*) with slight modifications. Accordingly, 200 μL of the extracts were added to 75 μL of 5 % NaNO_2_ and left to stand for 6 min. Then, 150 μL of 10 % AlCl_3_ were added and left to stand for another 5 min. Then 500 μL of 1 M NaOH were added. After 30 min of incubation in the dark at room temperature the absorbance was measured at 510 nm in a Multiskan Go microplate spectrophotometer (Thermo Fisher Scientific). Total flavonoid content was expressed in mg catechin equivalents (CE) per g of dry mass.

### Total antioxidant capacity (TEAC method)

The Trolox equivalent antioxidant capacity (TEAC) method was used to determine the antioxidant activity of the samples. The analysis was carried out according to Re *et al.* (*30*). Accordingly, a 7 mM ABTS^•+^ solution containing 2.45 mM potassium peroxydisulfate was prepared. The radical solution was diluted with phosphate-buffered saline (PBS) solution to give an absorbance value of (0.700±0.020) at 734 nm. Then, 1 mL of ABTS^•+^ solution was added to 10 μL of sample extract. After stirring gently for 6 min, the absorbance values were recorded. The percentile reduction ratio was calculated based on the initial absorbance value of the ABTS^•+^ solution. After 6 min, this value is called the inhibition ratio. This process was repeated twice, the inhibition ratios were calculated and their mean values were determined. Then, the same procedures were repeated by changing the sample volume (2.5, 5.0, 7.5 and 10.0 μL). Thus, the inhibition ratios of each sample and their mean values were determined. The sample concentrations corresponding to the sample amounts (volume) were then calculated. The inhibition ratio values were then plotted against the sample concentrations, a linear regression analysis was carried out, and the curve for the sample and the equation defining this curve were obtained. The TEAC value of the sample was calculated by proportioning the slope of the curve of the sample to the slope of the standard curve (*31*). Total antioxidant capacity was expressed as mM of Trolox equivalents (TE) per g dm.

### Total antioxidant activity (DPPH method)

The total antioxidant activity of the samples was determined using the 2,2-diphenyl-1-piprylhydrazyl (DPPH) radical scavenging method (*32*, *33*). To 100 µL of the extracts, 2 mL of 0.1 mM DPPH (1,1-diphenyl-2-picrylhydrazyl) were added and the tubes were mixed thoroughly. After 30 min at room temperature in the dark, the absorbance was measured at 517 nm using a Multiskan Go microplate spectrophotometer (Thermo Fisher Scientific). The results were expressed as inhibition percentage. The radical scavenging activity was calculated using the following equation:


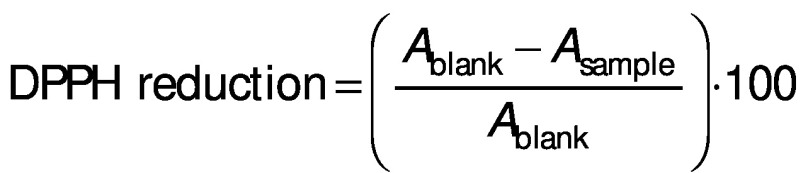
 /1/

### The qualitative analysis of phenolic compounds

An Agilent 6530 Accurate-Mass brand QTOF/LC-MS (Santa Clara, CA, USA) was used to qualitatively analyse the phenolic compounds in artichoke leaves. After the sample extraction with 50 % ethanol, dilutions of different concentrations were prepared using methanol (99.99 %). An electrospray ionisation source (ESI Dual Agilent jet stream) was used as the ion source in the analysis and worked in negative and positive modes as the ion mode. Poroshell 120 SB-C18 (3.0 mm×100 mm×2.7 µm; Agilent) was used as the analytical column. The injection volume was 5 µL and the flow rate was set to 0.6 mL/min. The gradient working programme used in the analysis consisted of water and methanol containing 5 mM ammonium acetate. The total analysis time was 30 min and the gradient operating conditions for LC-QTOF-MS were as follows: 0–25 min 95 % A, 5 % B; 25–28 min 5 % A, 95 % B; 28–30 min 95 % A, 5 % B, where A is 5 mM ammonium acetate and B is methanol.

Scanning was performed at a scanning speed of 2 spectra/s in the range of 100–1500 *m/z* ratio (monoisotopic mass) for the component analysis of the samples. The QTOF ionisation conditions were as follows: gas temperature 175 °C, drying gas flow rate 14 L/min, Fogger pressure 3102.6 MPa, sheath gas temperature 350 °C, sheath gas flow rate 11 L/min, fragmenter 300 V OCT 1 RF Vpp 750 V, cooler 65 V and sprayer 1000 V.

### The analyses of antimicrobial activity

The extracts obtained from artichoke leaves were dehydrated and then weighed. They were dissolved in 1 mL of 6 % dimethyl sulfoxide (Merck, Rahway, NJ, USA) and this solution was used for antimicrobial analyses.

Gram-positive (*Staphylococcus aureus* ATCC 6538, *Bacillus cereus* ATCC 10876, *Bacillus subtilis* ssp. *spizizenii* ATCC 6633, *Enterococcus faecalis* ATCC 29212 and *Listeria monocytogenes* NCTC 11994), Gram-negative (*Escherichia coli* ATCC 8739, *Enterobacter cloacae* ATCC 13047, *Salmonella enterica* serovar Typhimurium ATCC 14028 and *Pseudomonas aeruginosa* ATCC 15442) bacteria and one yeast species (*Candida albicans* ATCC 10231) were tested to determine the antimicrobial efficiency of artichoke extracts. Bacterial cultures were inoculated on trypticase soy agar (Merck) for 18–24 h at 37 °C (18–24 h at 30 °C for *Bacillus* cultures) and yeast cultures (*C. albicans*) were inoculated on Sabouraud dextrose agar (Merck) for 48 h at 30 °C. Before the experiments, the density of the culture suspensions was adjusted to 0.5 according to the McFarland standard turbidity using a densitometer (Grant, Cambridge, UK).

### Agar diffusion method

A volume of 100 µL of culture suspensions was inoculated on Mueller-Hinton agar (Merck) (for bacteria) and Sabouraud dextrose agar (for yeasts) and spread using a sterile L-shaped glass rod. After drying the surface of the media, the 8-mm wells were punched using the tip of a sterile pipette. Then, 60 μL of artichoke extracts were transferred to the wells and allowed to diffuse at room temperature for a maximum of 2 h. Ampicillin (10 μg/mL) and gentamicin (10 μg/mL) discs for bacteria were used as the positive control, while fluconazole (25 μg/mL) discs for *C. albicans* and discs impregnated with 6 % DMSO were used as the negative control. Mueller-Hinton agar plates were incubated at 37 °C for 18–24 h (30 °C for *Bacillus* cultures), while Sabouraud dextrose agar plates were incubated at 30 °C for 24–48 h and the diameters of the inhibition zones formed at the end of the incubation period were measured.

### Broth microdilution method

The minimum inhibitory concentration (MIC) values of artichoke leaf extracts were determined using the broth microdilution method (*34*). Accordingly, 80 µL of Mueller-Hinton broth for bacteria and Sabouraud dextrose broth for *C. albicans* were transferred to each well of the 96-well microplate. Then 80 µL of the extract were transferred and diluted 2-foldin the first well. Then, 20 μL of microorganism suspension were inoculated into all wells at a final amount of 10^6^ CFU/mL. The last two wells were used as positive and negative controls. Ampicillin (10 μg/mL) or gentamicin (10 μg/mL) for bacteria and fluconazole (25 μg/mL) solution for yeast were used as positive controls. The medium and culture mixtures were used as a negative control. After the lids were sealed, the inoculated microplates were incubated at a suitable temperature and time for each organism. At the end of incubation, 0.5 % of 2,3,5-triphenyltetrazolium chloride (Merck) was added to each well to determine growth. After 30 min of incubation, the wells with colour changes were evaluated as positive and the MIC value was determined.

### Statistical analysis

The SPSS v. 22.0 (*35*) package programme was used to statistically analyse the results of the samples. One-way analysis of variance and Duncan’s multiple range test with a 99 % confidence interval were performed. The results were calculated based on two replicates and two parallels.

## RESULTS AND DISCUSSION

### The design applied in the extraction methods

In all extraction methods, the temperature was standardised to (50±5) °C. Table 1 shows that the microwave-assisted extraction was used at two power values, 800 and 440 W (MAE1 and MAE2, respectively). In our preliminary experiments we determined extraction times of 45 and 30 s, respectively, to maintain the extraction temperature of (50±5) °C. The ultrasound-assisted extraction with probe was applied in two different modes, namely continuous and pulsed mode (50 % duty cycle) each for 30 and 15 min (UAEp1 to UAEp4). The ultrasound-assisted extraction in a water bath was also applied at the power of 240 and 120 W, the extraction times were 30 and 15 min at each power (UAEwb1 to UAEwb4). For maceration, extraction was performed in a shaking incubator at 50 °C for 24 and 12 h (M1 and M2).

**Table 1:** Extraction conditions of all extraction methods, total phenolic content (TPC), total flavonoid content (TFC) and antioxidant activity of the extracts of artichoke leaves

Sample code	Parameter	*t*/min	TPC as *w*(GAE)/(mg/g)	TFC as *w*(CE)/(mg/g)	TEAC as *b*(TE)/(mM/g)	DPPH inhibiton/%
MAE1	*P*=800 W	0.5	(3.72±0.03)^a^	(8.3±0.3)^a^	(57.9±2.4)^a^	(85.2±1.7)^a^
MAE2	*P*=440 W	0.75	(3.4±0.4)^a^	(6.50±0.01)^b^	(50.0±0.1)^bc^	(70.7±2.3)^e^
UAEp1	Continuous process	30	(2.72±0.00)^b^	(6.6±0.5)^b^	(51.4±4.2)^bc^	(84.1±2.5)^ab^
UAEp2	Continuous process	15	(2.4±0.5)^bc^	(6.1±0.1)^bc^	(42.2±6.5)^d^	(79.9±3.2)^cd^
UAEp3	Pulsed process (50 % duty cycle)	30	(2.7±0.6)^b^	(6.5±0.7)^b^	(52.0±0.8)^b^	(81.2±0.5)^bc^
UAEp4	Pulsed process (50 % duty cycle)	15	(2.48±0.04)^bc^	(5.6±0.3)^cd^	(46.2±1.8)^cd^	(77.4±1.7)^d^
UAEwb1	*P*=240 W	30	(2.5±0.6)^bc^	(5.3±0.2)^d^	(42.8±0.8)^d^	(69.4±1.3)^ef^
UAEwb2	*P=*240 W	15	(1.9±0.1)^c^	(3.2±0.4)^g^	(32.4±1.6)^e^	(66.4±1.4)^fg^
UAEwb3	*P*=120 W	30	(2.3±0.3)^bc^	(4.3±0.5)^e^	(32.1±2.2)^e^	(69.9±1.5)^ef^
UAEwb4	*P*=120 W	15	(1.9±0.5)^c^	(4.0±0.8)^efg^	(34.0±5.8)^e^	(63.6±2.6)^g^
M1	-	1440	(2.0±0.1)^c^	(4.0±0.1)^ef^	(43.3±1.9)^d^	(69.4±1.7)^ef^
M2	-	720	(1.89±0.02)^c^	(3.55±0.05)^fg^	(42.6±2.8)^d^	(60.0±1.7)^h^

All results are expressed as the mean value*±*standard deviation, *N*=4. Mean values within each column followed by different letters in superscript are significantly different at p≤0.01. MAE=microwave-assisted extraction, UAEp=ultrasound-assisted extraction with probe, UAEwb=ultrasound-assisted extraction in a water bath, M=maceration, GAE=gallic acid equivalents, CE=catechin equivalents, TE=Trolox equivalents

### Bioactive properties

Table 1 also shows the results for TPC, TFC and antioxidant capacity of the artichoke leaf samples extracted by different methods. The data set can be found in the supplementary material.

Microwave-assisted extraction was the most efficient among all extraction methods in terms of TPC. This could be due to the rapid interaction between the microwave energy and the solvent molecules, resulting in the rapid disintegration of the cell walls and easier release of the bioactive compounds (*17*) from artichoke leaves. No statistically significant results were found between MAE1 and MAE2 (p>0.01). It is known that microwave power is an important parameter to increase extraction efficiency by maximising the molecular interactions between the electromagnetic field and the sample (*36*). However, longer exposure time at lower microwave power, as in MAE2, may not result in any statistical difference between MAE1 and MAE2 samples. Exposure time is an important parameter to increase the efficiency of the microwave-assisted extraction, but long-term exposure at high microwave power can also degrade some phenolic compounds (*36*-*38*). Xia *et al.* (*37*) found that an extraction time of up to 10 min increased the oxymatrine extraction efficiency, but after 10 min the extraction efficiency decreased due to the degradation of plant material at long extraction times. In another study, microwave-assisted extraction of *Barleria lupulina* Lindl. at constant microwave power for 60 s yielded higher TPC than for 30 s (*36*).

Ultrasound-assisted extraction with probe was the second efficient method for TPC and showed the results, expressed as GAE on a dry mass basis, in the range of 2.40–2.72 mg/g. Similar to our results, Cheng *et al.* (*39*) found higher amounts of total phenolics in jackfruit pulp extracted by microwave-assisted extraction than by ultrasound-assisted extraction with probe with 60 % ethanol at constant extraction temperature. Both microwave-assisted and ultrasound-assisted extraction with probe are known to be effective in extracting phytochemicals from biological samples (*40*, *41*). However, in most studies, the extraction temperature is not standardised, which leads to different results in favour of microwave or ultrasound depending on the used parameters. In the ultrasound-assisted method, the type of process, whether continuous or pulsed, did not significantly affect the total phenolic content (p>0.01). This result was attributed to the use of a constant temperature in both extraction methods and the same result was obtained after both 30 and 15 min of extraction. Pan *et al.* (*42*) also investigated the continuous and pulsed ultrasound-assisted extraction of antioxidants from pomegranate peels and found high yields of total phenolics in both continuous and pulsed extraction (50 % of duty cycle). However, extraction times were significantly important in the ultrasound-assisted extraction (p≤0.01) and higher results were obtained with longer extraction times. Herrero *et al.* (*43*) reported that the extraction of phenolic compounds increased with extraction time at controlled temperature in ultrasound-assisted extraction. The increase in TPC with time in ultrasound-assisted extraction of artichoke by-products was also shown by Punzi *et al.* (*24*). The same results were obtained in ultrasound-assisted extraction in a water bath of artichoke leaves. While the results between the applications of the power of 240 and 120 W were not statistically significant (p>0.01), the effect of extraction time was statistically significant (p<0.01) as in ultrasound-assisted extraction with probe. Shortening the extraction time resulted in lower TPC than with the ultrasound-assisted extraction with probe. With the same extraction time, higher TPC were extracted with ultrasound-assisted extraction with probe than with the ultrasound-assisted extraction in a water bath. Ultrasound-assisted extraction in a water bath only operates at a single frequency, generally 20 or 40 kHz, while ultrasound-assisted extraction with probe provides a greater ultrasound power, at least up to 100 times stronger than that of the water bath. Moreover, the extraction in a water bath allows indirect sonication, which means that the ultrasonic waves must penetrate the wall of the sample container. In ultrasound-assisted extraction with probe, on the other hand, the probe is immersed directly into the sample to allow direct sonication in the sample (*12*, *44*). Sukor *et al.* (*44*) found that the efficiency of ultrasound-assisted extraction with probe was 15 % better than that of water bath extraction for the extraction of phenolic acids from *Quercus infectoria* galls. In our study, this value was 10–22.5 %.

Maceration was the least effective of all the applied methods, except for UAEwb2 and UAEwb4. Here, however, the time did not have a significant effect on the TPC (p>0.01). In solid-liquid extraction systems such as maceration, the release of bioactive compounds occurs according to the diffusion process until equilibrium is reached (*45*, *46*). Thus, the equilibrium of phenolic diffusion can be reached before or after 12 h during maceration.

In several studies, different TPC values were obtained in the extraction of artichoke leaves. Punzi *et al.* (*24*) found, on fresh mass basis, 1343 mg/kg of TPC in artichoke leaves extracted by methanol at room temperature for 60 min. Noriega-Rodríguez *et al.* (*19*) found the best result for TPC, expressed as GAE on dry mass basis, to be 2.16 g/100 g in hydroalcoholic extraction of artichoke waste, which is about 10 times more than our results. Awad *et al.* (*27*) also found the best result for TPC in microwave-assisted extraction of artichoke by-products on dry mass basis 193.63 µg/mg, which is about 52 times higher than our results. Jiménez-Moreno *et al.* (*26*) compared the efficiency of combined methods of ultrasound-assisted extraction in a water bath and maceration to maceration alone for TPC extraction from artichoke waste. These differences may be related to the applied extraction parameters (volume fraction of solvent, extraction time, temperature, method, *etc.*), the variety of the artichoke or other discarded parts of the artichoke.

Flavonoids are one of the most important polyphenols abundant in artichoke waste (*26*). MAE1 was the most efficient method for the extraction of TFC. Microwave-assisted extraction was found to be useful for the extraction of flavonoids in the literature (*41*, *47*). There was a statistically significant difference in the TFC between MAE1 and MAE2 (p≤0.01) but this difference was insignificant in the TPC. This result indicates the importance of applied power in microwave-assisted extraction of flavonoids (*47*) from artichoke leaves since it determines the absorption of microwave energy during extraction (*48*). We also speculated that the increase in extraction time could lead to degradation of flavonoids in artichoke leaves, as was also reported by Hithamani and Srinavasan (*49*). Li *et al.* (*50*) also found that increasing the microwave extraction time from 40 to 60 s significantly decreased the TFC, while this was not significant for the TPC of white sorghum.

Ultrasound-assisted extraction with probe was the second most effective and both the continuous and pulsed process for 30 min (UAEp1 and UAEp3) showed better results than both processes for 15 min (UAEp2 and UAEp4). The type of process, continuous or pulsed, did not significantly affect the extraction of TFC (p>0.01) as the extraction of TPC. This result shows the importance of the application of a constant temperature in both ultrasound-assisted extractions. The same result was obtained after both 30 and 15 min of extraction. However, the effect of extraction time was statistically significant (p≤0.01) and higher TFC was found after 30 min of extraction than TPC. The effect of extraction time on TFC was shown in several studies (*51*, *52*).

In ultrasound-assisted extraction in a water bath, the TFC, expressed as catechin equivalents (CE), ranged from (3.2±0.4) to (5.3±0.2) mg/g, with extractions UAEwb1 and UAEwb3 giving the best results and the extraction time also being statistically significant, as in ultrasound-assisted extraction with probe (p≤0.01), while the percentage of power was not statistically significant, just like the results for TPC in ultrasound-assisted extraction with probe.

Maceration was the least efficient of all applied methods, showing a TFC, expressed as CE, of (4.0±0.1) and (3.6±0.6) mg/g in M1 and M2, respectively. To the best of our knowledge, no previously published study has reported any TFC results in the extraction of artichoke leaves using different extraction methods. Only Jiménez-Moreno *et al.* (*26*) determined the efficiency of the combined methods of ultrasound-assisted extraction in a water bath and maceration compared to maceration alone for TFC in artichoke waste. In agreement with our results, Zeković *et al.* (*53*) investigated the extraction of sage by-products using different extraction methods. They reported higher TFC ​​of the samples processed using the ultrasound-assisted extraction with probe and microwave-assisted extraction than those of the samples that were macerated for 24 h in a shaking incubator.

It is reported that the antioxidant activity of artichoke leaves could be due to flavonoids (*54*) and according to our results, there was a significant positive correlation between TEAC and TFC (r^2^=0.795, p≤0.01). Moreover, the correlation between TEAC and TPC was also statistically significant (r^2^=0.732, p≤0.01). These results show that the total phenolic compounds and flavonoids make a major contribution to the antioxidant capacity of artichoke leaves. Also, the results of the phenolic compound qualitative analysis indicate that artichoke leaf samples are rich in polyphenols and flavonoids.

The results of antioxidant activity showed that the extraction MAE1 gave the highest results, expressed as Trolox equivalents (TE) on dry mass, in terms of TEAC and DPPH assay with (57.9±2.4) mM/g and (85.2±1.7) %. The results decreased to (50.0±0.1) mM/g and (70.7±2.3) % when the power was reduced to 440 W, and this difference was statistically significant (p≤0.01) as for the TFC. Ultrasound-assisted extraction with probe gave results, expressed as TE on dry mass basis, for antioxidant capacity ranging from (42.2±6.5) to (52.0±0.8) mM/g and (77.4±1.7) to (84.1±2.5) %. Statistical analysis of the results for TEAC showed no difference among MAE2, UAEp1, UAEp3 and UAEp4 treatments; UAEp2, UAEp4, UAEwb1, M1 and M2 treatments, and among UAEwb2, UAEwb3 and UAEwb4 treatments. When the DPPH values were statistically analysed, no difference was found between MAE1 and UAEp1 treatments, UAEp1 and UAEp3 treatments, and UAEwb1, UAEwb3 and M1 treatments. Although there were differences between the experimental results, no statistical difference was found. It was concluded that the time had a greater effect on antioxidant activity than the extraction method.

Both microwave-assisted and ultrasound-assisted extraction with probe are known to be efficient methods for the extraction of phytochemicals from biological samples (*40*, *41*). When applied to homogeneous suspensions, ultrasonication can induce cavitation and thus accelerate extraction. The components inside the cell can easily escape from the cell by breaking the cell wall (*40*). Microwave-assisted extraction uses high-frequency electromagnetic waves (300–300000 MHz), which lead to the disruption of the cell structure by the penetration of volumetric heating due to microwave irradiation (*15*, *16*). In our study, the extraction efficiency was found to be higher in microwave-assisted extraction and ultrasound-assisted extraction with probe because these methods disrupt the cell structure and extract the phytochemicals from the cell. Similar to our results, Cheng *et al.* (*39*) found similar ABTS^•+^ radical scavenging activity for the extracts from jackfruit pulp in both microwave-assisted extraction and ultrasound-assisted extraction with probe at constant extraction temperature. However, in most studies, the extraction temperature is not set to a standardised temperature, leading to different results in favour of microwave or ultrasound depending on the applied parameters. UAEp1 and UAEp3 showed higher results than UAEp2 and UAEp4, demonstrating the importance of extraction time for antioxidant activity as in the TPC and TFC values. The increase in antioxidant activity with time in ultrasound-assisted extraction with probe was also shown by Punzi *et al.* (*24*) for extracts from artichoke by-products. The UAEwb1 method applied at the power of 240 W for 30 min gave the highest value for TEAC, while the other applications of ultrasound-assisted extraction in a water bath were statistically lower than the UAEwb1 method. In the DPPH assay, UAEwb1 and UAEwb3 showed the highest results, and extraction time was a significant factor. Both M1 and M2 methods applied for 24 and 12 h, respectively, gave similar results to UAEwb1 method for the ABTS^•+^ assay. When the TEAC results of UAEwb1 and M1 and M2 treatments were examined, it was found that there was no statistical difference. Similarly, there was no difference in DPPH results between UAEwb1 and M1, but UAEwb1 and M2 were statistically different. For DPPH assay, extraction time was important, giving statistically different results (p≤0.01). As a result, the increase in treatment time led to an increase in antioxidant activity in both maceration and both ultrasound applications.

When the extraction activities were evaluated in four different treatments, it was found that the extraction efficiency improved with increasing power and time. Ibrahim *et al.* (*55*) stated that solubilisation could be due to the higher diffusivity of the solutes and the breakdown of the strong solute-matrix interaction caused by van der Waals forces, bonds and dipole forces of the solute molecules and the active sites on the hydrogen-increasing matrix. It is believed that these bonds weaken and break with power and time. There are different results in the literature for the antioxidant activities of extracts of artichoke waste (*24*, *26*, *27*, *39*). These differences may be related to the extraction parameters used (volume fraction of solvent, extraction time, extraction temperature, extraction method, *etc*.), the variety of artichoke or other discarded parts of the artichoke.

The phenolic profile of MAE1 sample was analysed because it gave the best results for biochemical analyses. The *m/z* (monoisotopic mass) values of phenolic compounds were used for qualitative determination. LC-QTOF molecular ion masses of phenolic compounds detected in waste artichoke leaves are shown in Table 2. A total of eighteen metabolites were determined, five of which were phenolic acids (four caffeoylquinic acid derivatives: chlorogenic acid, 1,3-di-*O*-caffeoylquinic acid (cynarin), 4,5-di-*O*-caffeoylquinic acid and 1-*O*-caffeoylquinic acid, and 4-hydroxyphenylpyruvic acid) and the others were flavonoids including luteolin and its derivatives (luteolin 4'-*O*-glucoside, luteolin 7-*O*-glucuronid), quercetin 3,7-dirhamnoside, quercetin 5,7,3',4'-tetramethyl ether 3-rutinoside, baicalein, 2''-*O*-alpha-l-rhamnosyl-6-C-fucosyl-luteolin, 4'-hydroxy-5,7,2'-trimethoxyflavanone 4'-rhamnosyl-(1-6)-glucoside, patuletin 3-(4''-acetylrhamnoside)-7-(3'''-acetylrhamnoside), scutellarein (6-hydroxyapigenin), 5,2',5'-trihydroxyflavone, 6-hydroxyluteolin 6-rhamnoside and chrysoeriol 6-C-glucoside-8-C-arabinopyranoside. It was determined that most of the phenolic compounds detected in our study were also found in different studies that used other methods of extraction of artichoke leaves. Uluad (*56*) found luteolin, chlorogenic acid, neochlorogenic acid, apigenin, naringin and kaempferol as the main compounds in artichoke leaves extracted under reflux with 70 % methanol. Jiménez-Moreno *et al.* (*26*) used combined methods of UAEwb and maceration to extract the inner and outer leaves and stems of artichokes with a 60 % methanol-water solution. They determined that the most abundant polyphenolic compounds were chlorogenic acid, luteolin-7-*O*-rutinoside and 7-*O*-glucoside. In addition, they also found low amounts of cynarin, luteolin, apigenin, apigenin-7-*O*-glucoside, apigenin-7-*O*-rutinoside and naringenin-7-*O*-glucoside. Farag *et al.* (*57*) determined the phenolic profile of artichoke leaves extracted with 50 % ethanol and found eight caffeic acid derivatives, six saponins, twelve flavonoids and ten fatty acids. Since the LC-QTOF-MS technique is a high-resolution technique that enables broad screening, comprehensive profiling and a large data library, it also allows the determination of a low amount of known and unknown metabolites. For this reason, there may be some differences in the phenolic profile of artichoke leaves, which could be due to the differences of the plant, *i.e.* the regions where the plant is grown, the extraction method used, the solvent used and the parameters of analysis.

**Table 2:** Phenolic composition of artichoke leaf waste

			Molecular	*M*_mi_	
No.	Phenolic compound	Type	formula	Determined	Actual
1	Chlorogenic acid	Phenolic acid	C_16_H_18_O_9_	353.08	354.09
2	Luteolin	Flavonoid	C_15_H_10_O_6_	285.04	286.04
3	Luteolin 4'-O-glucoside	Flavonoid	C_21_H_20_O_11_	447.09	448.10
4	Luteolin 7-O-glucuronide	Flavonoid	C_21_H_18_O_12_	461.07	462.07
5	Quercetin 3,7-dirhamnoside (polyphenol)	Flavonoid	C_27_H_30_O_15_	593.15	594.15
6	1,3-Di-*O*-caffeoylquinic acid (cynarine)	Phenolic acid	C_25_H_24_O_12_	515.12	516.12
7	Baicalein	Flavonoid	C_15_H_10_O_5_	269.04	270.05
8	4,5-Di-*O*-caffeoylquinic acid	Phenolic acid	C_25_H_24_O_12_	515.12	516.12
9	2''-*O*-alpha-l-rhamnosyl-6-C-fucosyl-luteolin	Flavonoid	C_27_H_30_O_14_	577.15	578.16
10	4-Hydroxyphenylpyruvic acid	Phenolic acid	C_9_H_8_O_4_	179.03	180.04
11	Patuletin 3-(4''-acetylrhamnoside)-7-(3'''-acetylrhamnoside)	Flavonoid	C_32_H_36_O_18_	707.18	708.19
12	4'-Hydroxy-5,7,2'-trimethoxyflavanone 4'-rhamnosyl-(1-6)-glucoside	Flavonoid	C_30_H_38_O_15_	683.22	638.22
13	Quercetin 5,7,3',4'-tetramethyl ether 3-rutinoside	Flavonoid	C_31_H_38_O_16_	665.20	666.21
14	Scutellarein (6-hydroxyapigenin)	Flavonoid	C_15_H_10_O_6_	285.03	286.04
15	1-O-caffeoylquinic acid	Phenolic acid	C_16_H_18_O_9_	353.086	354.09
16	5,2',5'-Trihydroxyflavone	Flavonoid	C_15_H_10_O_5_	269.04	270.05
17	6-Hydroxyluteolin 6-rhamnoside	Flavonoid	C_21_H_20_O_11_	447.09	448.10
18	Chrysoeriol 6-C-glucoside-8-C-arabinopyranoside	Flavonoid	C_27_H_30_O_15_	593.14	594.15

### Antimicrobial properties

Samples MAE1, UAEp1, UAEwb1 and M1 were determined to be the most effective representatives of each extraction method. Antimicrobial activity tests were carried out against Gram-positive (*Staphylococcus aureus* ATCC 6538, *Bacillus cereus* ATCC 10876, *Bacillus subtilis* ssp. *spizizenii* ATCC 6633, *Enterococcus faecalis* ATCC 29212 and *Listeria monocytogenes* NCTC 11994), Gram-negative (*Escherichia coli* ATCC 8739, *Enterobacter cloacae* ATCC 13047, *Salmonella enterica* serovar Typhimurium ATCC 14028 and *Pseudomonas aeruginosa* ATCC 15442) bacteria and a yeast species (*Candida albicans* ATCC 10231) using agar diffusion and broth microdilution methods. Table 3 and Table 4 show the results of the agar diffusion method and the MICs determined for these samples, respectively. The inhibition zone of the extracts on the microorganisms was determined using the agar diffusion method. The zone diameters for bacteria were in the range of 10.3–18.7 mm for MAE1, 9.8–22.0 mm for UAEp1, 8.3–16.3 mm for UAEwb1 and 8.0–14.0 mm for M1 sample. No antifungal activity was observed against *C*. *albicans.* Antibiotics ampicillin (10 μg/mL) or gentamicin (10 μg/mL) and fluconazole (25 μg/mL) were more effective than artichoke extracts.

**Table 3:** Antimicrobial activity of artichoke leaf extracts determined using agar diffusion method

	*d*(inhibition zone)/mm						
Tested microorganism	MAE1	UAEp1	UAEwb1	M1	Amp	Gen	Flu
*Staphylococcus aureus* ATCC 6538	(18.7±0.6)	(22.0±0.0)	(16.3±0.6)	(14.0±0.0)	(28.0±0.0)	-	-
*Bacillus cereus* ATCC 10876	(12.0±0.00)	(13.2±0.3)	(10.5±0.5)	(11.0±0.00)	-	(20.0±0.0)	-
*Bacillus subtilis* ssp. *spizizenii* ATCC 6633	(10.8±0.3)	(10.3±0.6)	(8.7±0.6)	(9.2±0.3)	-	(22.0±0.0)	-
*Enterococcus faecalis* ATCC 29212	(15.0±0.0)	(18.7±0.6)	(14.5±0.5)	(14.0±0.0)	(26.0±0.5)	-	-
*Listeria monocytogenes* NCTC 11994	(16.3±0.6)	(17.0±0.0)	(15.7±0.6)	(13.7±0.6)	-	(26.0±0.0)	-
*Escherichia coli* ATCC 8739	(15.5±0.5)	(15.7±0.6)	(12.2±0.3)	(12.0±0.0)	-	(23.0±0.5)	-
*Enterobacter cloacae* ATCC 13047	(14.0±0.0)	(13.3±0.6)	(11.3±0.6)	(11.7±0.3)	-	(22.0±0.2)	-
*Salmonella enterica* serovar Typhimurium ATCC 14028	(12.7±0.6)	(11.2±0.3)	(10.30±0.58)	(10.8±0.3)	-	(21.0±0.00)	-
*Pseudomonas aeruginosa* ATCC 15442	(10.3±0.3)	(9.8±0.3)	(8.30±0.58)	(8.0±0.0)	-	(18.0±0.5)	-
*Candida albicans* ATCC 10231	0.0	0.0	0.0	0.0	-	-	(26.0±0.0)

Results are expressed as mean value±standard deviation, *N*=4. Negative control (6 % DMSO)=0.0 mm. MAE1=microwave-assisted extraction, UAEp1=ultrasound-assisted extraction with probe, UAEwb1=ultrasound-assisted extraction in a water bath, M1=maceration, Amp=ampicillin, Gen=gentamicin, Flu=fluconazole

**Table 4:** Minimum inhibitory concentration (MIC) of artichoke leaf extracts

	*γ*(MIC)/(µg/mL)			
Tested microorganism	MAE1	UAEp1	UAEwb1	M1
*Staphylococcus aureus* ATCC 6538	50	25	100	100
*Bacillus cereus* ATCC 10876	50	50	100	100
*Bacillus subtilis* ssp. *spizizenii* ATCC 6633	50	50	100	100
*Enterococcus faecalis* ATCC 29212	50	25	50	50
*Listeria monocytogenes* NCTC 11994	50	50	100	100
*Escherichia coli* ATCC 8739	100	100	150	150
*Enterobacter cloacae* ATCC 13047	150	150	150	100
*Salmonella enterica* serovar Typhimurium ATCC 14028	50	100	100	100
*Pseudomonas aeruginosa* ATCC 15442	150	150	200	200

MAE1=microwave-assisted extraction, UAEp1=ultrasound-assisted extraction with probe, UAEwb1=ultrasound-assisted extraction in a water bath, M1=maceration

In this study, the MIC values of the extracts obtained using four different methods were determined in the range of 25–200 μg/mL. In the antimicrobial activity tests, applications of UAEp1 and MAE1 were found to have the largest zone diameter and the lowest inhibitory concentration due to their high values of TPC and TFC. The presence of phenolics in a substance can often lead to antimicrobial properties and act as a chemical defence mechanism against invading microorganisms. Conversely, flavonoids have been shown to inhibit bacterial growth by interfering with DNA function, disrupting cytoplasmic membrane performance and altering energy metabolism (*21*). Generally, the antibacterial efficacy against Gram-positive bacteria was higher, while the effects on spore-forming organisms were lower. The tested extracts had low activity against *P. aeruginosa*, which is resistant to many antibacterial agents due to its molecular resistance mechanisms.

The antimicrobial activity of extracts obtained from different parts of the artichoke, such as leaves, stems and rhizomes, has been investigated in several studies. Vamanu *et al.* (*58*) used samples obtained from the extraction with different ethanol volume fractions and reported 8–17 mm zone diameters formed against microorganisms in a diffusion study. Zhu *et al.* (*22*) investigated the changes in phenolic content and antimicrobial activity of artichoke leaves, stems and hearts by extraction with different solvents. They reported that the effective MIC values for the test organisms were between 1.25 and 10 μg/mL. Studies have shown that different parts of the artichoke, such as the heart, leaves and stem, have different amounts of phenolic content and that the antimicrobial activity increases proportionally with the increasing amount of phenolic content (*22*, *58*).

## CONCLUSIONS

This study showed the effect of different parameters of extraction methods known as green technologies to increase the extraction yield. Namely, microwave-assisted extraction at the highest power and constant extraction temperature was the most efficient method in terms of total phenolic and flavonoid content and antioxidant capacity of artichoke leaves, possibly due to the rapid interaction between the microwave energy and the solvent molecules, which led to a rapid release of bioactive compounds. This result was also important because the microwave-assisted extraction had the shortest extraction time of 30 s. It was also observed that as the microwave power increased, the yield of the analysed components increased. Ultrasound-assisted extraction with probe was another effective method, while ultrasound-assisted extraction in a water bath gave lower results than ultrasound-assisted extraction with probe due to indirect sonication. Whether the type of process was continuous or pulsed did not affect the results of either method, probably because a constant temperature was used for the same period of time. However, shortening the extraction time reduced the yields for both methods. Maceration yielded the lowest amount of biochemical compounds and showed the importance of using green technologies in the extraction of artichoke leaves. Furthermore, our qualitative analysis of the phenolic compounds revealed the presence of various phenolic acids and flavonoids in the artichoke leaf extracts, which contribute to their antioxidant and antimicrobial properties. Additionally, we found a significant positive correlation between the total phenolic, total flavonoid content and the antioxidant capacity of the extracts, emphasising their potential health benefits. In terms of antimicrobial activity, the extracts obtained from MAE1 showed high efficiency against many opportunistic and pathogenic microorganisms.

Overall, our findings suggest that MAE and UAEp methods are effective for obtaining bioactive compounds from artichoke leaves, which possess significant antioxidant and antimicrobial properties. These results could be valuable for the development of functional food products and pharmaceuticals with enhanced health benefits.

While this study has investigated the effect of different parameters for MAE, UAEp, UAEwb and maceration, further research should investigate fine-tuning these parameters to achieve even greater efficiency and yield by applying a variety of different analysis methods such as morphological analysis.
